# The Acute-Phase Ambulation Program Improves Clinical Outcome for Acute Heart Failure

**DOI:** 10.3390/jcdd9100314

**Published:** 2022-09-20

**Authors:** Yusuke Funato, Yuji Kono, Hideki Kawai, Meiko Hoshino, Akira Yamada, Takashi Muramatsu, Masahide Harada, Hiroshi Takahashi, Yohei Otaka, Masanobu Yanase, Hideo Izawa

**Affiliations:** 1 Department of Cardiology, Fujita Health University School of Medicine, Toyoake 470-1101, Japan; 2 Rehabilitation Complex, Fujita Health University Hospital, Toyoake 470-1192, Japan; 3 Department of Rehabilitation Medicine II, Fujita Health University School of Medicine, Toyoake 470-1101, Japan

**Keywords:** acute-phase ambulation program, cardiac rehabilitation, exercise, heart failure, secondary prevention

## Abstract

It remains unclear whether the acute-phase ambulation program (AAP) improves the prognosis of heart failure (HF) patients. We examined the association between the initiation of AAP and the prognosis of patients with worsening HF. We enrolled 560 consecutive patients admitted due to worsening HF from March 2019 to April 2021. Our hospital introduced AAP in May 2020, but we did not perform AAP until April 2020. We retrospectively compared cardiac events within 180 days after discharge between patients admitted before April 2020 (conventional group) and after May 2020 (AAP group). Primary endpoints were all-cause mortality and readmission for worsening HF. The Kaplan–Meier survival curves showed a significantly lower event rate in the AAP group in HF readmission or the primary endpoint (*p* = 0.020 and *p* = 0.014). The occurrence of the primary endpoint was associated with age, history of HF, systolic blood pressure, medications including renin–angiotensin system inhibitors or angiotensin receptor blocker, hemoglobin, NT-proBNP, and AAP participation. After adjusting for these parameters and sex, participation in AAP was an independent factor associated with a reduced risk of primary endpoint occurrence (hazard ratio of 0.62 (0.41–0.95), *p* = 0.028). The AAP for patients with acute HF might lead to improved short-term prognosis and should be considered for implementation.

## 1. Introduction

The number of patients with heart failure (HF) has increased with the aging of society [1], and the readmission rate for HF has not improved [2]. In patients with HF, the implementation of a guideline-based medical therapy reportedly reduced the incidence of cardiac deaths, but the rate of rehospitalization due to HF has not declined [3]. On the other hand, exercise therapy has been reported to be effective in various kinds of disease, including mental disorders and infectious diseases [4,5]. Accumulating evidence has shown that comprehensive cardiac rehabilitation for HF improves exercise tolerability, prevents readmission [6,7], and improves long-term prognosis through multiple mechanisms, including suppression of neurohumoral factors and inflammatory cytokines [8,9] and improvement of skeletal muscle metabolic dysfunction [10]. Recently, comprehensive cardiac rehabilitation, including exercise training, has been reported to improve the rate of readmission for HF, regardless of the left ventricular ejection fraction (LVEF) [7]. In Japan, comprehensive cardiac rehabilitation has been implemented based on the “Standard Cardiac Rehabilitation Program for Heart Failure” by the Japanese Society of Cardiac Rehabilitation [11]. This standard program recommends initiating the acute-phase ambulation program (AAP) at bedside immediately after hemodynamic stabilization, ideally leading to early implementation of exercise training. In this AAP, the therapist performs a loading test in the acute phase, and if the vitals are still stable, the degree of bed rest level is increased. We start with a low exercise load that requires only a sitting position, then gradually increase the exercise load, and increase the degree of bed rest level. In addition, for patients with severe heart failure or severe frailty, or for patients with a circulatory support device who have difficulty with even the weakest exercise load, low-intensity resistance training, bed-based rehabilitation, and neuromuscular electrical stimulation are performed to suppress the progress of disuse [11]. However, the effectiveness of AAP has not been fully evaluated. We hypothesized that AAP could improve the postdischarge cardiovascular events in patients with HF. Therefore, the aims of the present study were to examine the association between the initiation of AAP and the prognosis of patients with worsening HF and to investigate the relationship between AAP implementation and postdischarge cardiovascular events in patients with HF.

## 2. Methods

### 2.1. Patients 

This single-center retrospective study enrolled consecutive patients who were admitted due to worsening HF from March 2019 to April 2021 in Fujita Health University Hospital, a sophisticated hospital in Toyoake City, Japan. The study protocol was approved by the institutional review board of Fujita Health University (identifier: HM20-161), which waived the requirement for obtaining patients’ written informed consent because of the retrospective study design.

**Figure 1:**
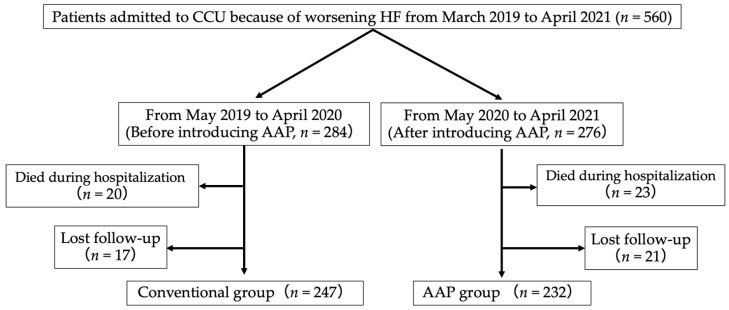
Study flow diagram.

At our hospital, we implemented AAP in all patients admitted due to worsening HF since May 2020. A total of 560 patients were admitted to our hospital from May 2019 to April 2021. Patients admitted between May 2020 and April 2021 who participated in AAP were assigned to the AAP group (*n* = 284), whereas those hospitalized between May 2019 and April 2020, when AAP had not been implemented yet, were assigned to the conventional group (*n* = 276). From each group, we excluded those who died and could not be followed up for 180 days. Thus, 247 and 232 patients in the AAP and conventional groups, respectively, were included in the final analysis (Figure 1).

### 2.2. Primary Events and Clinical Characteristics

The primary outcomes were rehospitalization due to worsening HF and all-cause mortality. Cardiovascular events within 180 days after discharge were evaluated in the outpatient medical record of each patient.

We reviewed the medical records of all patients to evaluate demographic data, such as age, sex, body mass index (BMI), HF causes, medications, and comorbidities. Echocardiography and blood sampling were performed on the day of admission. LVEF was calculated using the modified Simpson’s rule on echocardiography at admission. The total protein, hemoglobin, and plasma N-terminal pro-brain natriuretic peptide (NT-proBNP) levels were measured for all patients.

### 2.3. Statistical Analysis

We used the unpaired Student’s *t*-test or Mann–Whitney *U* test to compare continuous variables, expressed as mean ± standard deviation (SD) or median (interquartile range (IQR)) due to a non-normal distribution, as appropriate. Categorical variables are presented as numbers or percentages and were compared using a chi-squared test. Univariate and multivariate Cox regression analyses were used to evaluate predictive capability. The Cox regression analysis involved a predictive model based on pre-existing potential prognostic and confounding factors. This model used log NT-proBNP, hemoglobin, BMI, LVEF, chronic kidney disease (CKD), and history of hospitalization for HF as adjusting variables. We conducted Kaplan–Meier analysis with a log-rank test to compare the rates of readmission due to worsening HF between the AAP and conventional groups. All analyses were performed using JMP (JMP Pro ver. 15.1.0), and *p* < 0.05 was considered significant. 

## 3. Results

### 3.1. Patients’ Clinical Characteristics and Primary Events

The baseline patient characteristics are listed in Table 1. Compared with the conventional group, the AAP group showed a greater BMI and a higher heart rate. Mean age, sex distribution, and medical history were similar between the AAP and conventional groups. The prescription rates of the sodium–glucose transporter 2 (SGLT2) inhibitor and the angiotensin receptor–neprilysin inhibitor (ARNI) were higher in the AAP group than in the conventional group. There were no significant differences in hemoglobin and the total protein levels between the two groups. There were no significant differences between the two groups for intravenous diuretics (conventional group: 94% vs. AAP group: 92%) and dobutamine administration during hospitalization (conventional group: 22% vs. AAP group: 26%). All patients’ medications remained unchanged during the postdischarge follow-up period. The rate of hospitalization due to HF was higher in the conventional group than in the AAP group.

### 3.2. Survival Analyses

**Figure 2:**
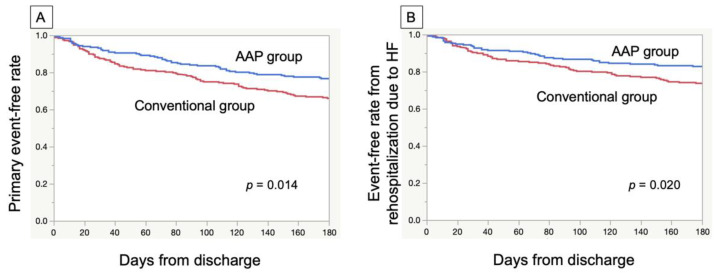
Kaplan–Meier curves for the period to a cardiac event. Subjects were divided into the AAP and conventional groups. (**A**) Primary composite endpoint. (**B**) Rehospitalization due to HF. AAP = acute-phase ambulation program; HF = heart failure.

The Kaplan–Meier survival curves after the log-rank test showed a significantly lower event rate in the AAP group than in the conventional group (Figure 2).

Furthermore, only readmission was analyzed as an event, and the event avoidance rate was significantly higher in the AAP group than in the conventional group.

### 3.3. Univariate and Multivariate Analyses

Table 2 shows the results of the Cox regression analysis for the primary event. In the univariate analysis, AAP participation was found to be a significant related factor associated with the primary event. Multivariate analysis was performed after adjustment for predictors in the univariate analysis of our study or in the previous studies. In the multivariate analysis that included age, sex, history of HF, systolic blood pressure, medications including renin–angiotensin system inhibitors or angiotensin receptor blockers, hemoglobin, NT-proBNP, and AAP participation as regulators, AAP participation was found to be a significant independent factor associated with the primary endpoint.

## 4. Discussion

We retrospectively examined the association between the initiation of AAP and all-cause mortality and readmission due to worsening HF within 180 days after discharge in 479 patients hospitalized for acute HF. Our findings showed that, in our hospital, the cardiovascular events might have been suppressed after the AAP initiation, as compared with before its initiation.

The introduction of the mobility protocol, which provides exercise training to patients based on a program, such as AAP, has been reported to reduce the length of hospital stay [12] and improve the patients’ ability to perform activities of daily living (ADLs) at discharge [13] by implementing the protocol on noncardiac patients using the respiratory system in the intensive care unit (ICU). However, the relationship between the initiation of the mobility protocol during hospitalization in HF patients and the prognosis of these patients after discharge has not been confirmed. Only one previous study reported that the initiation of mobility protocols during hospitalization of patients with HF improved the rate of readmission due to HF. However, in this previous study, the postdischarge follow-up duration was short, and a multivariate analysis was not performed [14]. For the first time, we report the association between the initiation of AAP during hospitalization and the improvement of the prognosis of patients with HF after discharge.

One of the reasons for the improvement in the prognosis of patients with HF after initiating AAP during hospitalization could be the maintenance of muscle strength. Decreased skeletal muscle strength increases the ergoreceptor activity, which influences the activity of sympathetic and neurohumoral factors, which leads to shortness of breath and exacerbation of HF. In addition, this mechanism itself causes endocrine abnormalities, peripheral circulatory insufficiency, inflammation, and oxidative stress, resulting in a vicious cycle that leads to a decrease in skeletal muscle strength [9]. Furthermore, a history of long-term HF results in a decrease in type I muscle fibers and an increase in type IIb muscle fibers, which cause anaerobic metabolism; tachypnea appears due to lactic acidosis, which may be a mechanism for the exacerbation of HF [10]. In a previous study, quadriceps muscle strength was greater in ICU patients without HF within 5 days of admission who received aerobic exercise training daily for 20 minutes (training group (1.83 ± 0.91 vs. 2.37 ± 0.62 N·kg^−1^, *p* < 0.01) versus control group (1.86 ± 0.78 vs. 2.03 ± 0.75 N·kg^−1^, *p* < 0.11). Moreover, the 6-minute walk distance at discharge was also reported to be improved in this previous study (196 vs. 143 m, *p* < 0.05) [15]. Moreover, Hülsmann et al. showed that the group with stronger knee flexors had a better prognosis by comparing the 5-year survival rates of patients with reduced EF using a cutoff of 68 Nm·100/kg for muscle strength [16]. The results of these previous studies indicate that early mobilization can maintain the muscle strength during hospitalization, which could subsequently improve the prognosis of patients with HF. This mechanism supports the findings of our study.

Another reason for improving the prognosis in patients with HF participating in AAP during hospitalization could be to remind physicians to prescribe exercises at discharge. AAP can easily lead to exercise prescription at discharge. Kamiya et al. reported that, in their multicenter study, the incidence of cardiovascular events was reduced in patients with HF, irrespective of EF, who received exercise training three to five times a week within 3 months after discharge according to Cox proportional hazards analysis (hazard ratio (HR), 0.77 (95% confidence interval, 0.65–0.92); *p* = 0.003) [7]. Thus, we assume that AAP could improve the rate of exercise training based on exercise prescription after discharge, leading to an improvement in patients with HF.

Finally, improving the prognosis in patients with HF participating in AAP during hospitalization could be useful in educating patients on self-management interventions for HF by a multidisciplinary team. AAP can easily lead to education of self-management interventions by a multidisciplinary team. Jonkman et al. reported that the incidence of HF-related readmission and all-cause mortality during 6 months after discharge was decreased when self-management interventions based on the education provided by a multidisciplinary team were implemented in addition to exercise training [17]. In this study, not all patients reached the sixth stage during the implementation of this AAP. It is possible that AAP may have improved the prognosis of patients with HF through mechanisms other than the action on the skeletal muscle described above.

### Study Limitations

There are several limitations to this study. First, patients in this study did not receive optimal medicine therapy. The prescription rates of ARNI or the SGLT2 inhibitor in this study were low, because these drugs were not available in patients with HF in Japan until recently. In addition, the prescription rates of the angiotensin-converting enzyme inhibitor, angiotensin receptor blocker, beta-blocker, and mineralocorticoid receptor antagonist were low, as well as ARNI and SGLT2, because this study included many elderly patients, reflecting Japan’s aging society, and these elderly patients often had hypotension and renal failure [18]. Then, the results of this study could not be applicable to the current HF population in other countries. In addition, the prescription rates of ARNI and the SGLT2 inhibitor were higher in the AAP group than in the conventional group, but the association between these drugs and event occurrence was not significant in the COX regression analysis.

Second, we could not analyze the muscle mass and the duration of HF education and postdischarge exercise training implemented for the patients. Third, cardiopulmonary exercise testing was not performed in all patients before and after hospitalization in this study. VO2 max by cardiopulmonary exercise testing is considered useful for prognostic evaluation of heart failure [19], and VO2 max could be a possible target in future prospective studies. Finally, the present study was a single-center retrospective investigation, and the possibility of confounding factors cannot be ruled out.

## 5. Conclusions

In this study, we found the association between the initiation of AAP and all-cause mortality and readmission in patients with HF. Thus, in the acute phase of HF, it may be advisable to initiate a mobility protocol to improve the short-term prognosis of patients.

## Figures and Tables

**Table 1:** Patient characteristics.

	Conventional Group (*n* = 247)	AAP Group (*n* = 232)	*p*-Value
Age (IQR)	78 (70–85)	78 (70–84)	0.808
Male (%)	149 (60)	143 (61)	0.768
Ischemic cardiomyopathy (%)	51 (20)	47 (20)	0.917
Atrial fibrillation (%)	124 (50)	96 (41)	0.052
Hypertension (%)	168 (68)	164 (70)	0.526
Diabetes (%)	97 (39)	89 (38)	0.838
Dyslipidemia (%)	102 (41)	93 (40)	0.788
Chronic kidney disease (%)	195 (78)	172 (74)	0.214
History of hospitalization due to Heart failure (%)	122 (49)	93 (40)	0.041 *
BMI (IQR)	22.2 (19.6–25.3)	23.1 (20.8–25.6)	0.045 *
SBP at admission (IQR)	147 (124–169)	142 (123–164)	0.561
HR at admission (IQR)	102 (85–119)	95 (80–115)	0.028
LVEF (IQR)	36 (27–51)	40 (30–54)	0.063
Serum hemoglobin (IQR)	11.7 (10.2–13.3)	11.6 (9.8–13.4)	0.835
Serum total protein (IQR)	6.4 (5.9–6.8)	6.4 (5.9–6.8)	0.998
NT-proBNP (IQR)	5991 (2884–12371)	5491 (2932–13449)	0.804
Medicine at discharge (%)			
ACE inhibitor or ARB	115 (47)	123 (53)	0.158
Beta blocker	171 (69)	155 (67)	0.570
Diuretics	204 (83)	187 (80)	0.575
Statin	95 (38)	98 (42)	0.399
MRA	153 (62)	130 (56)	0.189
SGLT2 inhibitor	10 (4)	33 (14)	0.001 *
ARNI	0 (0)	5 (2)	0.007 *

*p* value *, statistically significant (*p* < 0.05). AAP = acute-phase ambulation program; HF = heart failure; BMI = body mass index; SBP = systolic blood pressure; HR = heart rate; LVEF = left ventricular ejection fraction; NT-proBNP, N-terminal pro-brain natriuretic peptide; ACE = angiotensin converting enzyme; ARB = angiotensin receptor blocker; MRA = mineralocorticoid receptor antagonist; SGLT2 = sodium–glucose transporter 2; ARNI = angiotensin receptor–neprilysin inhibitor.

**Table 2:** Univariate and multivariate Cox regression hazard models for rehospitalization and all-cause death in patients who participated in AAP.

	Hazard Ratio	*p*
Univariable	0.58 (0.39–0.88)	0.010 *
Multivariable	0.62 (0.41–0.95)	0.028 *

*p* value *, statistically significant (*p* < 0.05). Adjusted models: adjusted for age, sex, history of hospitalization due to heart failure, systolic blood pressure, medications including renin–angiotensin system inhibitors or angiotensin receptor blockers, hemoglobin, NT-proBNP, and AAP participation. AAP = acute-phase ambulation program; NT-proBNP = N-terminal pro-brain natriuretic peptide.

## Data Availability

Data are stored in the hospital’s secure database.
